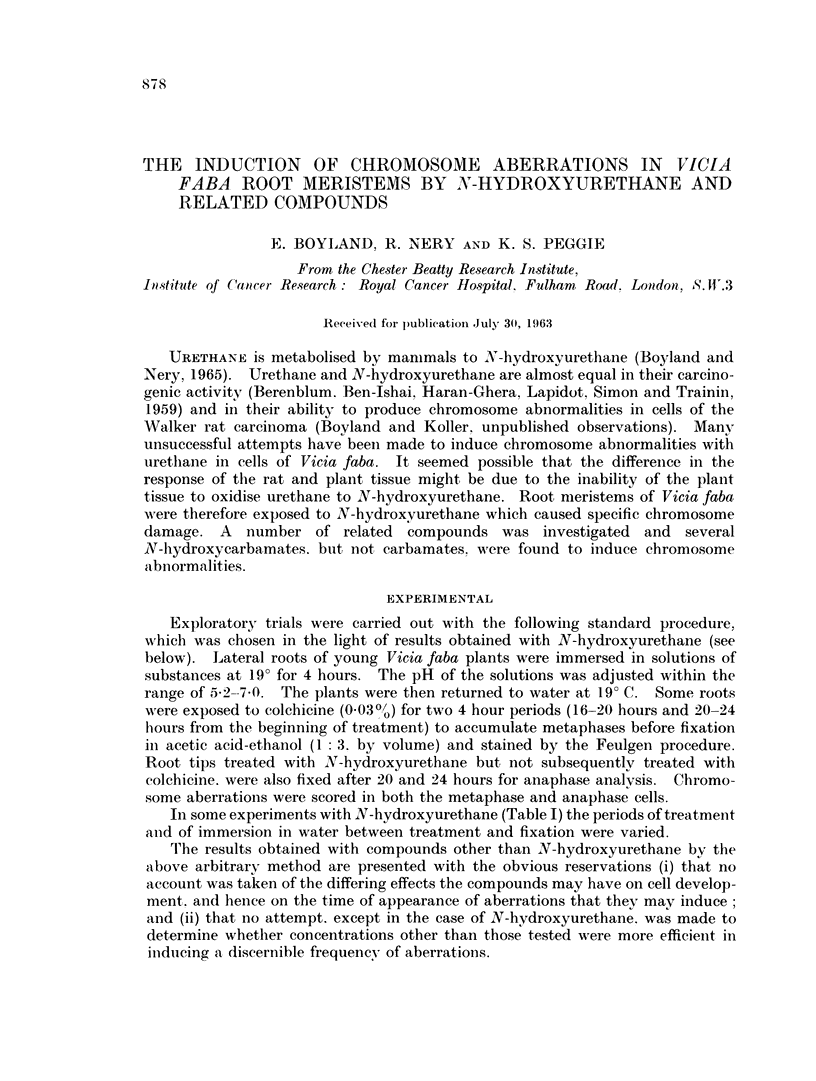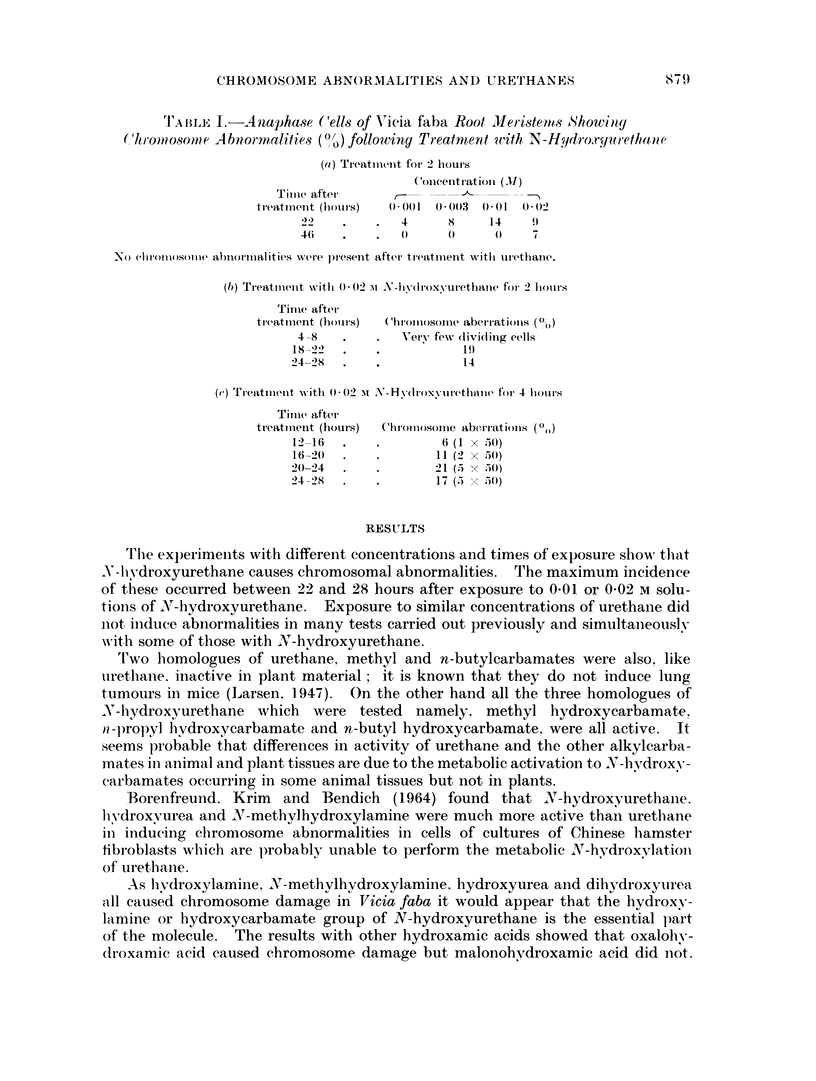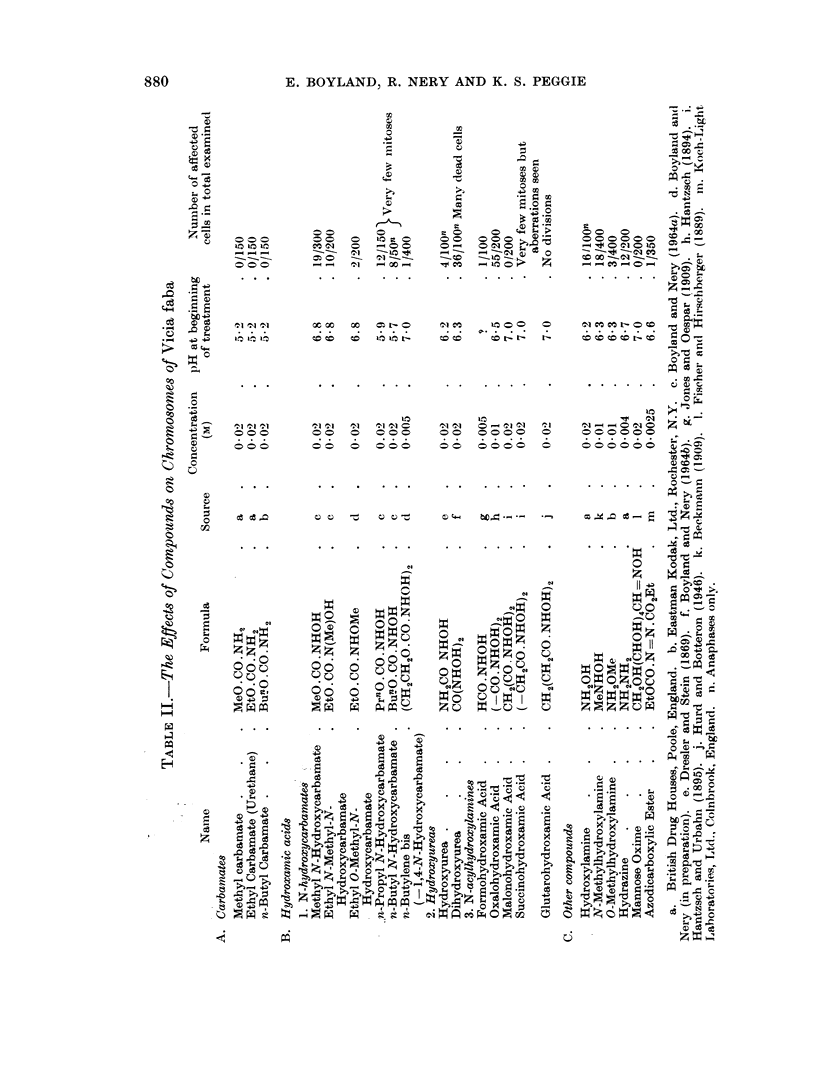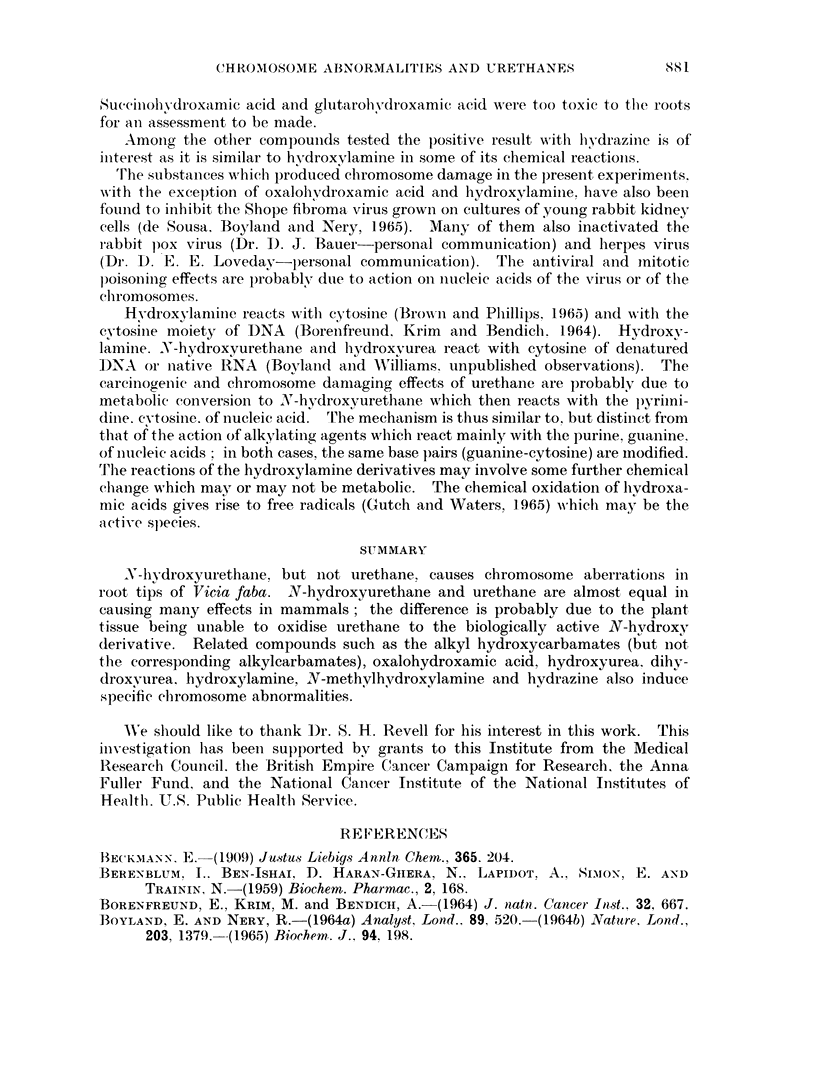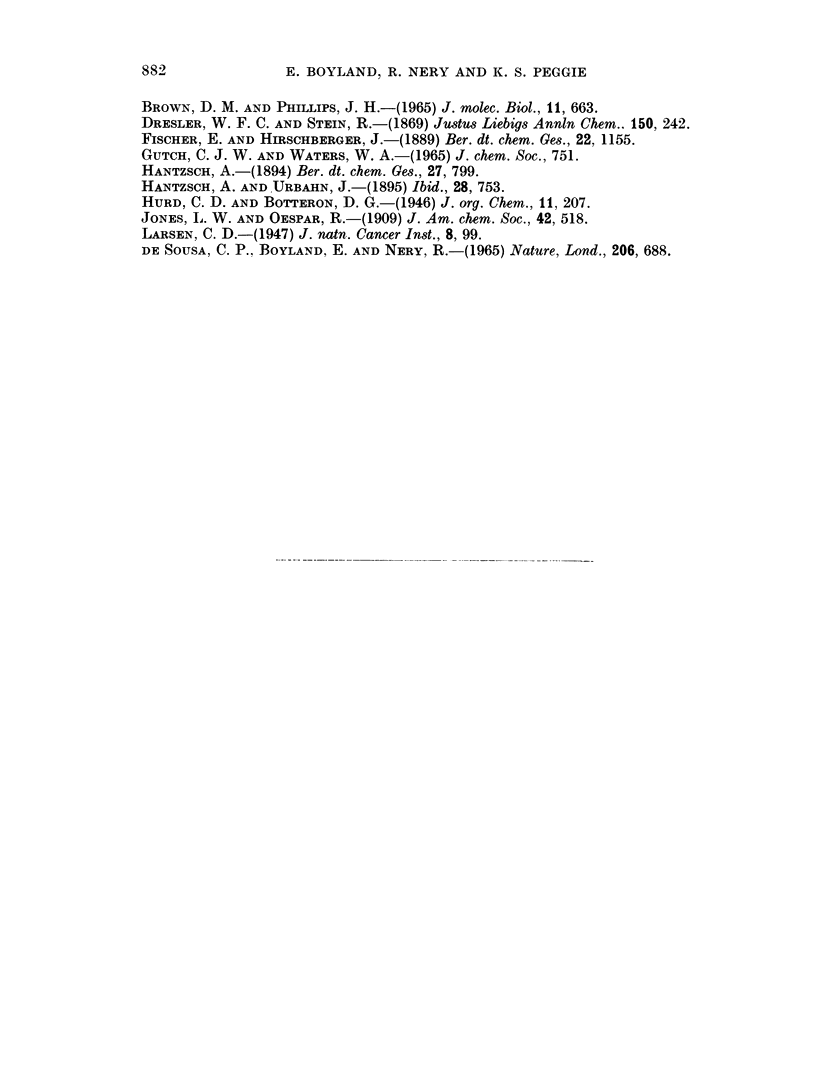# The induction of chromosome aberrations in Vicia faba root meristems by N-hydroxyurethane and related compounds.

**DOI:** 10.1038/bjc.1965.102

**Published:** 1965-12

**Authors:** E. Boyland, R. Nery, K. S. Peggie


### THE INDUCTION OF CHROMOSOME ABERRATIONS IN *VICIA FABA* ROOT MERISTEMS BY *N*-HYDROXYURETHANE AND RELATED COMPOUNDS

E. BOYLAND, R. NERY AND K. S. PEGGIE

*From the Chester Beatty Research Institute, Institute of Cancer Research: Royal Cancer Hospital, Fulham Road, London, S.W.3*

Received for publication July 30, 1963

URETHANE is metabolised by mammals to *N*-hydroxyurethane (Boyland and Nery, 1965). Urethane and *N*-hydroxyurethane are almost equal in their carcinogenic activity (Berenblum, Ben-Ishai, Haran-Ghera, Lapidot, Simon and Trainin, 1959) and in their ability to produce chromosome abnormalities in cells of the Walker rat carcinoma (Boyland and Koller, unpublished observations). Many unsuccessful attempts have been made to induce chromosome abnormalities with urethane in cells of *Vicia faba*. It seemed possible that the difference in the response of the rat and plant tissue might be due to the inability of the plant tissue to oxidise urethane to *N*-hydroxyurethane. Root meristems of *Vicia faba* were therefore exposed to *N*-hydroxyurethane which caused specific chromosome damage. A number of related compounds was investigated and several *N*-hydroxycarbamates, but not carbamates, were found to induce chromosome abnormalities.

### EXPERIMENTAL

Exploratory trials were carried out with the following standard procedure, which was chosen in the light of results obtained with *N*-hydroxyurethane (see below). Lateral roots of young *Vicia faba* plants were immersed in solutions of substances at 19° for 4 hours. The pH of the solutions was adjusted within the range of 5·2–7·0. The plants were then returned to water at 19° C. Some roots were exposed to colchicine (0·03%) for two 4 hour periods (16–20 hours and 20–24 hours from the beginning of treatment) to accumulate metaphases before fixation in acetic acid-ethanol (1 : 3, by volume) and stained by the Feulgen procedure. Root tips treated with *N*-hydroxyurethane but not subsequently treated with colchicine, were also fixed after 20 and 24 hours for anaphase analysis. Chromosome aberrations were scored in both the metaphase and anaphase cells.

In some experiments with *N*-hydroxyurethane (Table I) the periods of treatment and of immersion in water between treatment and fixation were varied.

The results obtained with compounds other than *N*-hydroxyurethane by the above arbitrary method are presented with the obvious reservations (i) that no account was taken of the differing effects the compounds may have on cell development, and hence on the time of appearance of aberrations that they may induce; and (ii) that no attempt, except in the case of *N*-hydroxyurethane, was made to determine whether concentrations other than those tested were more efficient in inducing a discernible frequency of aberrations.

TABLE I.—*Anaphase Cells of* Vicia faba *Root Meristems Showing Chromosome Abnormalities* (%) *following Treatment with* N-*Hydroxyurethane*

(*a*) Treatment for 2 hours

| Time after treatment (hours) | Concentration (*M*) | | | |
|---|---|---|---|---|
| | 0·001 | 0·003 | 0·01 | 0·02 |
| 22 . . | 4 | 8 | 14 | 9 |
| 46 . . | 0 | 0 | 0 | 7 |

No chromosome abnormalities were present after treatment with urethane.

(*b*) Treatment with 0·02 M *N*-hydroxyurethane for 2 hours

| Time after treatment (hours) | Chromosome aberrations (%) |
|---|---|
| 4–8 . . | Very few dividing cells |
| 18–22 . . | 19 |
| 24–28 . . | 14 |

(*c*) Treatment with 0·02 M *N*-Hydroxyurethane for 4 hours

| Time after treatment (hours) | Chromosome aberrations (%) |
|---|---|
| 12–16 . . | 6 (1 × 50) |
| 16–20 . . | 11 (2 × 50) |
| 20–24 . . | 21 (5 × 50) |
| 24–28 . . | 17 (5 × 50) |

### RESULTS

The experiments with different concentrations and times of exposure show that *N*-hydroxyurethane causes chromosomal abnormalities. The maximum incidence of these occurred between 22 and 28 hours after exposure to 0·01 or 0·02 M solutions of *N*-hydroxyurethane. Exposure to similar concentrations of urethane did not induce abnormalities in many tests carried out previously and simultaneously with some of those with *N*-hydroxyurethane.

Two homologues of urethane, methyl and *n*-butylcarbamates were also, like urethane, inactive in plant material; it is known that they do not induce lung tumours in mice (Larsen, 1947). On the other hand all the three homologues of *N*-hydroxyurethane which were tested namely, methyl hydroxycarbamate, *n*-propyl hydroxycarbamate and *n*-butyl hydroxycarbamate, were all active. It seems probable that differences in activity of urethane and the other alkylcarbamates in animal and plant tissues are due to the metabolic activation to *N*-hydroxycarbamates occurring in some animal tissues but not in plants.

Borenfreund, Krim and Bendich (1964) found that *N*-hydroxyurethane, hydroxyurea and *N*-methylhydroxylamine were much more active than urethane in inducing chromosome abnormalities in cells of cultures of Chinese hamster fibroblasts which are probably unable to perform the metabolic *N*-hydroxylation of urethane.

As hydroxylamine, *N*-methylhydroxylamine, hydroxyurea and dihydroxyurea all caused chromosome damage in *Vicia faba* it would appear that the hydroxylamine or hydroxycarbamate group of *N*-hydroxyurethane is the essential part of the molecule. The results with other hydroxamic acids showed that oxalohydroxamic acid caused chromosome damage but malonohydroxamic acid did not.

Succinohydroxamic acid and glutarohydroxamic acid were too toxic to the roots for an assessment to be made.

Among the other compounds tested the positive result with hydrazine is of interest as it is similar to hydroxylamine in some of its chemical reactions.

The substances which produced chromosome damage in the present experiments, with the exception of oxalohydroxamic acid and hydroxylamine, have also been found to inhibit the Shope fibroma virus grown on cultures of young rabbit kidney cells (de Sousa, Boyland and Nery, 1965). Many of them also inactivated the rabbit pox virus (Dr. D. J. Bauer—personal communication) and herpes virus (Dr. D. E. E. Loveday—personal communication). The antiviral and mitotic poisoning effects are probably due to action on nucleic acids of the virus or of the chromosomes.

Hydroxylamine reacts with cytosine (Brown and Phillips, 1965) and with the cytosine moiety of DNA (Borenfreund, Krim and Bendich, 1964). Hydroxylamine, *N*-hydroxyurethane and hydroxyurea react with cytosine of denatured DNA or native RNA (Boyland and Williams, unpublished observations). The carcinogenic and chromosome damaging effects of urethane are probably due to metabolic conversion to *N*-hydroxyurethane which then reacts with the pyrimidine, cytosine, of nucleic acid. The mechanism is thus similar to, but distinct from that of the action of alkylating agents which react mainly with the purine, guanine, of nucleic acids ; in both cases, the same base pairs (guanine-cytosine) are modified. The reactions of the hydroxylamine derivatives may involve some further chemical change which may or may not be metabolic. The chemical oxidation of hydroxamic acids gives rise to free radicals (Gutch and Waters, 1965) which may be the active species.

### SUMMARY

*N*-hydroxyurethane, but not urethane, causes chromosome aberrations in root tips of *Vicia faba*. *N*-hydroxyurethane and urethane are almost equal in causing many effects in mammals ; the difference is probably due to the plant tissue being unable to oxidise urethane to the biologically active *N*-hydroxy derivative. Related compounds such as the alkyl hydroxycarbamates (but not the corresponding alkylcarbamates), oxalohydroxamic acid, hydroxyurea, dihydroxyurea, hydroxylamine, *N*-methylhydroxylamine and hydrazine also induce specific chromosome abnormalities.

We should like to thank Dr. S. H. Revell for his interest in this work. This investigation has been supported by grants to this Institute from the Medical Research Council, the British Empire Cancer Campaign for Research, the Anna Fuller Fund, and the National Cancer Institute of the National Institutes of Health, U.S. Public Health Service.

### REFERENCES

BECKMANN, E.—(1909) *Justus Liebigs Annln Chem.*, **365**, 204.

BERENBLUM, I., BEN-ISHAI, D. HARAN-GHERA, N., LAPIDOT, A., SIMON, E. AND TRAININ, N.—(1959) *Biochem. Pharmac.*, **2**, 168.

BORENFREUND, E., KRIM, M. and BENDICH, A.—(1964) *J. natn. Cancer Inst.*, **32**, 667.

BOYLAND, E. AND NERY, R.—(1964*a*) *Analyst, Lond.*, **89**, 520.—(1964*b*) *Nature, Lond.*, **203**, 1379.—(1965) *Biochem. J.*, **94**, 198.

BROWN, D. M. AND PHILLIPS, J. H.—(1965) *J. molec. Biol.*, **11**, 663.
DRESLER, W. F. C. AND STEIN, R.—(1869) *Justus Liebigs Annln Chem.*. **150**, 242.
FISCHER, E. AND HIRSCHBERGER, J.—(1889) *Ber. dt. chem. Ges.*, **22**, 1155.
GUTCH, C. J. W. AND WATERS, W. A.—(1965) *J. chem. Soc.*, 751.
HANTZSCH, A.—(1894) *Ber. dt. chem. Ges.*, **27**, 799.
HANTZSCH, A. AND URBAHN, J.—(1895) *Ibid.*, **28**, 753.
HURD, C. D. AND BOTTERON, D. G.—(1946) *J. org. Chem.*, **11**, 207.
JONES, L. W. AND OESPAR, R.—(1909) *J. Am. chem. Soc.*, **42**, 518.
LARSEN, C. D.—(1947) *J. natn. Cancer Inst.*, **8**, 99.
DE SOUSA, C. P., BOYLAND, E. AND NERY, R.—(1965) *Nature, Lond.*, **206**, 688.